# Botanic Gardens Complement Agricultural Gene Bank in Collecting and Conserving Plant Genetic Diversity

**DOI:** 10.1089/bio.2018.0028

**Published:** 2018-10-12

**Authors:** Katherine O'Donnell, Suzanne Sharrock

**Affiliations:** Botanic Gardens Conservation International, Richmond, United Kingdom.

**Keywords:** botanic gardens, PGRFA, seed banking, *ex situ* collections, Crop Wild Relatives

## Abstract

Originating in Europe in the 16th century, botanic gardens are found in nearly every country in the world. Botanic gardens have had various roles during this time, including as physic gardens, as adaptation centers for commercial crops, as pleasure gardens, and more recently as conservation institutions. The role of botanic gardens in the conservation of Crop Wild Relatives is becoming increasingly important. At least 6000 taxa related to 68 crop genera are maintained by the world's botanic gardens with several gardens having specialist collections. The extent of infra-specific genetic diversity of these Plant Genetic Resources for Food and Agriculture (PGRFA) being conserved is currently unknown, although based on existing evidence it is likely to be low. However, these PGRFA collections, through display and education, play a significant role in linking the public to important issues, including food security and the need to conserve crop diversity. Today there are some 2700 botanic gardens in existence, and they are visited by over 500 million people every year. The majority of these gardens are found in temperate regions. At least 30% of all known plant species are found in botanic garden collections, including 41% of threatened species. These *ex situ* collections are conserved in a variety of ways, including as seed bank collections. Around 350 botanic gardens together maintain seed collections of 57,000 taxa. These seed bank collections have a variety of uses, including for research and reintroduction.

## Introduction

Botanic gardens are widely distributed around the world, with almost every country having at least one such garden. Botanic gardens are diverse institutions, but can be distinguished from amenity parks and other gardens by their scientific and educational focus. A generally recognized definition of a botanic garden is:

an institution holding documented collections of living plants for the purposes of scientific research, conservation, display, and education.^1^

Gardens and the cultivation of plants have been around for thousands of years, with the first examples dating back around 3000 years to ancient Egypt and Mesopotamia. In China gardens are known to have been developed during the Shang (1600–1046 BC) and Zhou (1045–256 BC) dynasties, and the Romans were also keen gardeners and aware of the medicinal properties of plants. In the Americas, the gardens of Texcotzingo, Mexico were developed by the Aztecs in the 15th century to collect and display plant (and animal) specimens, as well as the cultivation of medicinal plants. In Europe, the first “botanic” gardens are considered to be the physic gardens of Italy dating from the 16th and 17th centuries. The first of these physic gardens was the garden of the University of Pisa which was created by Luca Ghini in 1543. Other Italian universities rapidly followed suit, and gardens were created in Padova (1545), Firenze (1545), and Bologna (1547). These gardens were purely for the academic study of medicinal plants. By the 17th century these medicinal gardens had spread to universities and apothecaries throughout central Europe such as Cologne and Prague. The University of Oxford botanic garden was the first physic garden to be established in the United Kingdom in 1621.

Botanic gardens then experienced a change in usage during the 17th and 18th centuries. This was the age of exploration and the beginnings of international trade. Gardens such as the Royal Botanic Gardens (RBG), Kew and the Real Jardín Botánico de Madrid were set up to introduce and cultivate new species that were being brought back from expeditions to tropical, as well as temperate, regions. Not only did these gardens promote and encourage botanical exploration but also they helped find new gardens in tropical regions to help cultivate newly discovered plant species. An important function of these tropical gardens was to receive and cultivate commercial crops such as cloves, tea, coffee, breadfruit, cinchona, palm oil, and cocoa. During the 19th and 20th century, there was huge interest in the great variety of plants being discovered—including many that became prominent in horticulture. At this time, many municipal and civic gardens were created throughout Europe and the British Commonwealth. The Missouri Botanical Garden was among the first botanic gardens to be established in the United Kingdom in 1859, and the U.S. Botanic Garden has been continuously operating and open to the public since 1850, and its plant collection was established and has been maintained since 1842.

In recent decades, botanic gardens have developed a greater focus on science, as well as horticulture and education, largely due to the emergence of the conservation movement. They play an important role in conservation and habitat and species restoration due to their existing collections and the scientific knowledge they possess in the identification and propagation of plant species.

There are currently over 2700 botanic gardens and arboreta in countries around the world, with many more under construction or being planned. Indeed, in the last 50 years there has been an unprecedented growth in the establishment of botanic gardens, with over 60% of the gardens in existence today having been established since the mid-20th century.

The growing popularity of botanic gardens is also reflected in increasing visitor numbers, and it is estimated that today over 500 million people visit botanic gardens each year. Such gardens therefore play an important role in education and awareness raising, helping to instill a love of plants among the public and generating support for plant conservation.

## Botanic Garden Plant Collections

Botanic Gardens Conservation International (BGCI) is a worldwide networking organization, linking botanic gardens around the world. BGCI collects and collates information on the activities and facilities in botanic gardens and makes this available through its GardenSearch^*^ database. BGCI also compiles data on the astonishing array of plant diversity that is maintained in botanic garden plant collections and provides this information through its PlantSearch^†^ database. The database presently holds over 1.3 million records, representing 536,000 taxa, provided by around 1000 institutions.

An analysis of PlantSearch data was carried out recently.^2^ This study found that at least 30% of all known plant species (>105,000 species) are found in the *ex situ* collections of botanic gardens, including 41% of known threatened species. However, the study also showed that botanic gardens are disproportionally temperate, with 93% of species being held in the northern hemisphere and 76% of the species that are absent from collections being tropical in origin. Among the wide diversity of plant species maintained by botanic gardens are many that are likely to be of great importance in ensuring future food security.

## Botanic Gardens and Plant Genetic Resources for Food and Agriculture

The conservation of Plant Genetic Resources for Food and Agriculture (PGRFA) was once seen as exclusively the domain of crop gene banks. While such gene banks continue to play a leading role in conserving the wide diversity of local varieties and landraces of our major crops, the role of botanic gardens in the conservation of wild plant diversity related to cultivated crops (Crop Wild Relatives—CWR) is becoming increasingly important. In a survey of botanic garden staff from 2013, 69% of respondents considered “conserving local crop varieties and crop wild relatives” as a “very important” or “important” activity for botanic gardens.^3^

Achieving global food security while reconciling with demands on the environment is considered by many to be one of the greatest challenges facing humankind today. By 2050 it is likely that we will need to feed 9 billion people, with the increasing population also demanding a more varied protein-rich diet. All this means we need to grow more food on less land, with limited access to water and increasing costs for fertilizer and fuel. Developing improved crop varieties with enhanced resistance to pests, diseases, and environmental stress is key to developing a food system that has a lower impact on biodiversity and uses less land and water. Producing such varieties relies on the deployment of genes often found in the CWR and other wild plants maintained in botanic garden collections.

The conservation of plant genetic diversity is recognized as a key element in the achievement of Goal 2 of the United Nation's Sustainable Development Goals (SDGs). SDG 2 aims to “end hunger, achieve food security and improved nutrition, and promote sustainable agriculture,” and subtarget 2.5 is, by 2020: “Maintain genetic diversity of seeds, cultivated plants, … and their related wild species, including through soundly managed and diversified seed and plant banks at national, regional, and international levels, …”

This is closely related to Target 9 of the Convention on Biological Diversity's (CBD) Global Strategy for Plant Conservation, which calls for:

70 per cent of the genetic diversity of crops, including their wild relatives and other socioeconomically valuable plant species conserved, while respecting, preserving, and maintaining associated indigenous knowledge.^4^

An indication of the number of CWR and other species relevant to food and agriculture that are held within the living collections and seed banks of botanic gardens can be obtained by querying BGCI's PlantSearch database. This indicates that at least 6000 taxa related to 68 crop genera are maintained by the world's botanic gardens. This is likely a minimum number because PlantSearch only contains records for around one-third of the world's botanic gardens. Furthermore, PlantSearch does not give any indication of the number of accessions per taxa held by institutions, and in general such collections focus on high levels of species diversity, but often with little infra-specific diversity maintained. This contrasts with the crop gene banks, which generally have higher levels of within species diversity—large number of land races and local cultivars, but tend to be relatively poor in CWR. Table 1 provides information on the number of taxa (excluding cultivars) for selected crop genera held in botanic garden collections round the world. No distinction is made between living collection and seed bank data. We also recognize that the number of taxa may be exaggerated due to the lack of identification of synonyms used across collections. However, even when only accepted names (according to matches with names in the Plant List) are used, taxa numbers are high. For example, botanic garden collections maintain at least 585 distinct taxa of *Allium* and 400 taxa of *Solanum*.

**Table 1.** Number of Different Taxa Recorded in PlantSearch for Selected Crop Genera

*Genus*	*No. of taxa in collections*^a^	*Status of species name (accepted/synonym/unresolved*^b^*)*	*No. of institutions holding collections*	*No. of taxa in more than one botanic garden collection*
*Allium*	979	585/118/269	369 in 52 countries	483
*Capsicum*	46	22/12/12	119 in 41 countries	25
*Dioscorea*	138	98/17/23	226 in 47 countries	65
*Hordeum*	64	32/20/12	115 in 31 countries	37
*Ipomoea*	217	114/34/69	217 in 52 countries	119
*Phaseolus*	67	41/16/10	78 in 28 countries	26
*Solanum*	715	400/78/237	303 in 58 countries	288

^a^ Taxa include all with species names and also those with infra-specific rank (var./subspecies), but excluding cultivars.

^b^ According to matching again the PlantList.

It can also be seen that collections of each genus are spread over a large number of institutions and countries. For example, 369 institutions in 52 countries have collections of *Allium*, and 303 institutions in 58 countries include *Solanum* in their collections.

Having the same taxon maintained in multiple collections may provide an indication of the extent of diversity within that taxon that is conserved. The more collections that maintain a particular taxon, the more likely greater diversity will be conserved. For example, 182 institutions maintain the species *Allium schoenoprasum* and 125 institutions have *Allium tuberosum* in their collections. Further investigation of the origins of these accessions would be needed to confirm the extent of infra-specific diversity represented by these collections.

At the other end of the spectrum however are taxa that are only maintained in one collection. For the genera listed, it can be seen that this is the case for approximately half of all taxa. At the extreme, this might mean that only a few individuals of a particular taxa are being held in a single living collection for display purposes only. This material is therefore unlikely to be of any use for conservation or breeding purposes. However, these collections are still important from an educational and horticultural perspective, with knowledge of how to grow and propagate the taxon likely to be held within the institution.

Some botanic gardens have very extensive *ex situ* collections of particular crop species. For example, the Botanical and Experimental Garden, Radboud University, Belgium has a very significant collection of *Solanum*—consisting of nearly 400 taxa, while the Oekologisch-Botanischer Garten Universitaet Bayreuth, Germany maintains over 100 *Allium* taxa.

Other botanic gardens have specialized collections of PGRFA. These include the breadfruit collection at the Breadfruit Institute at the National Tropical Botanic Garden in Hawaii and the mango collection at the Fairchild Tropical Botanic Garden, which maintains more than 600 mango cultivars.

Case study: The conservation of *Malus* diversity Central Asia and Kazakhstan are the source of many cultivated plants, including garlic, apricots, and apples. The Institute of Botany and Phytointroductions located in the Botanic Garden, Almaty, Kazakhstan has played a particular role in the preservation and conservation of the wild apple, *Malus sieversii*. The cultivated apple (*Malus pumila* Mill.) is economically the fourth most important fruit crop in the world; it is nutritionally valuable and has been demonstrated through genomics to be related to *M. sieversii*. Unfortunately, the wild apple orchards of the Tien Shen mountains have been substantially destroyed through carelessness and by hybridization with domesticated apples.

The Institute and associated Botanic Garden have collected over 200 wild apple accessions which are being preserved *ex situ* from forest locations that are being conserved *in situ*. Over a period of about 40 years the apples have been gathered together to form an impressively diverse collection of materials. This collection is also being used to study the concentrations of biologically active substances present in the leaves and fruits and to provide clonal materials for forest restoration. It has been found that the concentrations of phenolics in the wild apples are, perhaps not surprisingly, much higher than in present day domesticated apples, but vitamin C levels are also substantially higher.^5^

While many PGRFA collections in botanic gardens are maintained as living collections or field gene banks, an increasing number of botanic gardens also have seed banks that include accessions of PGRFA. In Europe, a detailed analysis of the seed bank holdings of members of the European Native Seed Conservation Network (ENSCONET) Consortium has shown that 688 of the 913 species (75.36%) included on a combined checklist of CWR and medicinal plants are held in these collections.^6^ However, as is often the case with living collections, further analysis of the data showed that the infra-specific diversity of some of the collections was low, with 466 species being represented by less than four accessions.

## Seed Banking in Botanic Gardens

Botanic gardens maintain their *ex situ* plant collections in a variety of ways (Table 2). Seed banking offers a type of *ex situ* collection that can, if the correct protocols are followed, maintain high genetic diversity and high longevity at a relatively low cost (around £2,000 per species^8^). In addition, less space is required to maintain large samples than living collections. The majority of plant species produce orthodox seeds that can be stored in the seed bank. However, not all seeds can be stored in this way. Nonorthodox species cannot withstand drying and storage at low temperatures. For these species, cryopreservation with liquid nitrogen at −196°C is an option.

**Table 2.** Types of Plant Conservation Utilized by Botanic Gardens

*Type of* ex situ *collection*	*Genetic diversity*	*Longevity*	*Relative costs per individual*	*Relative conservation value*	*Notes*	*Examples of institutions applying this method and plant group*
Seed bank	High (if proper protocols followed)	High (with proper storage)	Low (if facilities exist)	Research—high reintroduction—high education—low	Seed storage is not possible for some species	Boyce Thompson Arboretum Desert Legume Program Orthodox species (e.g., Legumes)
Cryopreservation	High (if proper protocols followed)	High (with proper storage)	Intermediate (if facilities exist)	Research—high reintroduction—high education—low	Techniques for many species not yet available	Royal Botanic Gardens, Kew Recalcitrant species (e.g., Oaks)
Tissue culture	High (if proper protocols followed)	Intermediate (with proper storage)	Intermediate (if facilities exist)	Research—high reintroduction—high education—low	Techniques for many species not yet available	Cincinnati Zoo and Botanical Garden Orchids
Conservation collection/field gene bank	Intermediate	Short (species' generation time)	High	Research—high reintroduction—intermediate education—high	Cultivation is the only option for some species, adaptation to cultivation and hybridization is a concern	Xishuangbanna Tropical Botanic Garden Palms
Reference living collection	Low^a^	Short (species' generation time)	High	Research—intermediate reintroduction—low^a^ education—high	Source may be unknown, often one or few individuals, likely adaptation to cultivation	Ghent University Botanic Garden Begonias
Display living collection	Low^a^	Short (species' generation time)	High	Research—low^a^ reintroduction—low^a^ education—high	Source often unknown, often one or few individuals, likely adaptation to cultivation	Montgomery Botanical Center Cycads

Adapted from Kramer et al., 2011.^7^

^a^ May have higher genetic diversity or conservation and research value if material is wild-collected and maintained as multiple genetically diverse accessions, although adaptation to cultivation and hybridization is a concern.

Seed banking has traditionally been utilized by agricultural institutions focused on conserving crop diversity. Interest in using seed banks to conserve wild plants is however more recent. Spain was one of the first countries to focus on collection of wild flora, creating its seed bank in 1966, at the Department of Vegetal Biology of the Polytechnical University in Madrid. Several Spanish botanic gardens then created seed banks focused on conserving wild flora in the regions they were based (including the Jardín Botánico Canario Viera y Clavijo, Jardi Botanic de Soller Mallorca, Jardín Botánico de Córdoba, and at the Jardí Botànic de la Universitat de València). Together 10 of these seed banks formed the Spanish Network of gene banks for wild plants (Red Española de Bancos de Germoplasma de Plantas Silvestres) in 2002.^9^

An increasing number of botanic gardens are adopting seed banking as a way to conserve wild plant species, with the number of seed banks in botanic gardens having doubled in the last 20 years. The botanic garden community has some of the largest and most sophisticated seed banks but equally important are the numerous small-scale seed banks holding national or regional collections. According to BGCI's GardenSearch Database, there are at least 350 botanic gardens involved in seed banking in 74 countries.

Botanic gardens hold taxonomically and geographically diverse seed collections. Based on data from BGCI's PlantSearch database, there are in the region 57,000 taxa stored as seed bank collections representing nearly 7000 genera. The genera with the highest number of species in collections include *Acacia*, *Carex*, and *Eucalyptus*. Seed banks are present in 74 countries; however, their combined collections include endemic species from at least 166 countries.

One of the most important seed banks for wild plants, the RBG Kew's Millennium Seed Bank (MSB) opened in the year 2000 and through its partnership now represents the largest *ex situ* conservation program in the world. Together 96 partners aim to collect and conserve 25% of the world's orthodox species by 2020.^10^ Currently MSB holdings represent nearly 40,000 species.^11^

Examples of national efforts to conserve plant diversity through seed banking include the Korean National Arboretum Seed Bank (KNASB) in South Korea, which has during the past decade been involved in collecting and conserving wild, medicinal, and ornamental species with the aim of securing 60% of native Korean Species by 2020.^12^ KNASB collections are duplicated at the recently constructed Baekdudaegan National Arboretum Seed Vault, Asia's first and largest permanent seed store.^13^

Similarly, recent collaborative efforts in Hungary have seen agricultural institutions collaborate with various partner institutions, including the National Botanic Garden of Vácrátót, to set up the Pannon Seed Bank Project, which between 2010 and 2014 collected more than 60% of the endangered Hungarian flora.^14^

### The conservation role of seed banks

Seed banks act as a repository for species that are extinct in the wild. There are around 500 species found in seed banks in botanic gardens that are known to be regionally or globally extinct in the wild. An example includes the Rwandan *Nymphaea thermarum*. Overexploitation of the hot spring that feeds its habitat has led to the extinction of this species in the wild.

As the threat of invasive pests and diseases increases, seed bank collections can ensure the conservation of genetic diversity that may be eroded through disease in wild habitats. The fungal disease Myrtle Rust is now starting to have a serious impact on a number of species within the family Myrtaceae in New Zealand and Australia. Botanic gardens in both countries are involved in increased efforts to collect and bank seeds of affected species. Efforts to conserve seed bank collection of species threatened by invasive species are taking place in several other countries. In Hawai'i the Rapid Ōhi'a Death Seed Banking Initiative involves the Harold L. Lyon Arboretum collecting seed of the Ōhi'a tree. In the United Kingdom the MSB's UK Native Tree Seed Project is working to establish the first national collection of tree seeds, including *Fraxinus excelsior* (recently red listed as Near Threatened due to impeding threats from invasive species^15^). These seed bank collections can also be utilized as a research tool to determine disease susceptibility and resilience.

### BGCI and seed banking

Seed banking botanic gardens vary greatly in infrastructure, aims, and expertise. BGCI launched the Global Seed Conservation Challenge (GSCC) in 2014 to support seed banking in botanic gardens and to increase the number of threatened species in seed banks. The GSCC has five aims as follows:

- Engage more botanic gardens to become involved in seed banking, working “outside the garden walls” to bring into *ex situ* collections threatened species that are not already conserved;
- Highlight and celebrate success for seed conservation, including awarding prizes to gardens that excel;
- Strengthen networks to help botanic gardens share experiences and resources in seed banking;
- Establish a seed collecting “hub” at BGCI which will provide a “one-stop shop” for seed banking information and training resources;
- Provide training and build capacity to support seed collecting and raise seed banking standards.

BGCI also acts as Secretariat to the International Union for Conservation of Nature Species Survival Commission (SSC) Specialist Group on Seed Conservation. The chairs of the group include representatives from Harold L. Lyon Arboretum and the National Tropical Botanic Garden in the United States and Guangxi University in China. The creation of a Specialist Group aims to provide: (1) a network for the interchange of experiences and knowledge sharing in different ecosystems around the world, (2) the identification of regional and ecosystem gaps in *ex situ* seed conservation and aid in prioritization according to vulnerability, and (3) the creation of conservation and restoration policies according to the CBD.

As a first step of the network, an online directory of expertise was created^‡^ and currently includes over 400 individuals from over 300 institutions representing forestry, botanic garden, horticulture, and agricultural sectors. Included in this directory are the facilities that these institutions have, highlighting the capacity they have for conserving seed bank collections using the correct procedures and protocols.

### Seed banking protocols

Protocols used for collecting and banking seeds are important to ensure high quality seed of conservation value. However, there are no universally agreed protocols used by botanic gardens when collecting and banking seed. Instead, several protocols are used widely within the botanic garden community. Examples include those developed by the ENSCONET consortium, RBG Kew's Millennium Seed Bank Partnership (MSBP), and the U.S. Department of the Interior Bureau of Land Management's Seeds of Success program. These protocols incorporate the principles taken from the International Plant Genetic Resources Institute guidelines for collecting plant genetic diversity^16^ although they are adapted to wild flora collection, as there are fundamental differences present between crop and wild species. For example, crop species exhibit favored traits, such as uniformity of seed maturation. Wild collected seed however may flower and produce seed sporadically. This can lead to a collection being more complex and a variety of maturities being present within a seed collection. Several recent studies have focused on how to collect effectively to ensure high genetic diversity and additionally how to maintain genetic diversity within seed collections.^17–19^

To maintain quality collections, orthodox seeds need to be stored at low temperature and humidity. There are various standards used by botanic gardens. For example, the RBG, Kew has published seed banking standards focusing on the minimum requirements to ensure high quality seed collections containing recognized guidelines for seed storage, −20°C ± 3°C and 15% equilibrium relative humidity ±3%.^20^ Details of how botanic gardens implement best practice for long-term conservation of orthodox seeds by following these standards are available in BGCI's Botanic Gardens Manual.^21^ A survey of botanic garden seed banks suggests that not all seed banks follow these standards but that the majority of accessions are stored following recognized protocols.^22^

Seed banking in botanic gardens has led to an increase in research on wild species. These *ex situ* research studies of seed traits are an essential component to determining: if seed of a particular species can be stored in the seed bank (seed storage behavior), how long a species can be stored in the seed bank (longevity), if seeds are alive (seed viability), and how to overcome seed dormancy. Research findings related to these questions are essential to ensure survival and propagation of these seeds, ensuring that seed bank collections can be turned back into plants. Seed trait data are widely available. The RBG Kew's Seed Information Database^23^ holds, for example, 10,647 records on seed storage behavior comprising data from the Compendium of Information on Seed Storage Behavior^24^ and subsequent research carried out by the MSBP. In addition, the TRY Plant Trait Database^25^ has data on 60 traits related to seeds, including germination rate, seed viability, and germination requirement.

### Using seed bank collections

Seed collections are essential tools for conservation action, especially for research and for supporting *in situ* conservation in a species' natural habitat.

Botanic gardens have a long history of exchanging seed for a range of purposes, including for research, conservation, and display. Many gardens produce an annual “Index seminum” that provides information on the seeds they have available for exchange. These are usually either collected locally from the wild or collected from plants in their collections. Such seed exchanges can involve large numbers of transactions. For example, the Bonn Botanic Garden in Germany receives an average of 3600 orders for seed from its *index seminum* each year.

Similarly, Kew's MSB provides seed samples from their collection to institutions that are interested in using them. Data on use suggest that 12% of the taxa stored have been distributed for use in research, conservation, education, or display.^11^

The exchange of seed material by botanic gardens is governed by the principles of the CBD and particularly the Access and Benefit Sharing regulations as provided through the Nagoya Protocol. Seed exchange is generally only for noncommercial use and covered by Material Transfer Agreements (MTAs) that stipulate how seed can be used and how benefits from such use should be shared. The International Plant Exchange Network has been developed to provide a common framework for seed exchange for noncommercial use between participating botanic gardens, using a standard MTA.^§^

Seed bank collections can also be used to support *in situ* conservation. One such example is the work of the Harold L. Lyon Arboretum Seed Conservation Laboratory, of the Hawaiian Rare Plant Program. Along with other partners, they have been working to propagate seed bank collections to restore the extinct *Silene perlmanii* to a population of nearly 200 individuals.

There is a need for a strategic approach to habitat restoration, as nearly two-thirds of ecosystems worldwide are degraded.^26^ Although seed bank collections are used by botanic gardens as part of individual species reintroductions, there has been a call for these collections to shift from “stamp collections” of species. To ensure that, collections are able to provide the tons of seeds required for large-scale restoration and reintroduction.^27^ The SDGs and the United Nations' have a target to restore 15% of the world's degraded ecosystems by 2020.^28^ Recent initiatives working to address this target include the Ecological Restoration Alliance of Botanic Gardens (ERA), which aims to mobilize botanic gardens, arboreta, and seed banks to carry out science-based ecological restoration and the Society for Restoration's International Network for Seed Based Restoration, which aims to engage a wide range of stakeholders to foster understanding and advancement of seed-based restoration.

The Australian Botanic Garden, Mount Annan, a member of the ERA is one example of a botanic garden that is making this shift. A 1500 square meter native grass seed production area has been established at the garden, to provide seed specifically for restoration of cleared African olive sites. The first year output resulted in 118 kg of seed material harvested comprising 13 million viable seeds.

## Conclusions

Botanic gardens have a wealth of experience in conserving plant genetic resources. They have used the experience of crop gene banks and adapted working practices for the conservation of wild species. Their collections focus on wild plants and include a large number of CWR, making them very complementary to agricultural gene banks. Furthermore, with their comprehensive educational experiences and large visitor numbers, botanic gardens have the potential to play an important role in outreach and engaging the public in issue around crop diversity conservation and the origin of food crops.

Botanic garden seed banks include some of the world's most sophisticated infrastructures. These institutions are working to determine the best way to conserve the species of importance at the national, regional, or global level. However, it is essential that botanic garden collections be of sufficient quality and quantity to ensure that they are of use. In terms of conserving PGRFA, botanic gardens will never match the scope of the large gene banks of cultivated plants. However, the plants in their collections can play a valuable role in connecting people to the origins of their food crops and in preserving and conserving potentially valuable traits. Who knows what uses they might have in the future?

## Notes

^*^ www.bgci.org/garden_search.php

^†^ www.bgci.org/plant_search.php

^‡^ www.bgci.org/plant-conservation/seedexpertise

^§^ www.bgci.org/policy/ipen
